# Phenotype Switching in Pediatric Inflammatory Skin Diseases During Biologic Therapy: A Literature Review Focused on Atopic Dermatitis and Psoriasis

**DOI:** 10.3390/ijms27198740

**Published:** 2026-09-30

**Authors:** Chu-Fei Tsai, Yu Yu

**Affiliations:** 1 Department of Dermatology, Cathay General Hospital, Taipei City 106, Taiwan; 2 Department of Dermatology, Sijhih Cathay General Hospital, New Taipei City 221037, Taiwan

**Keywords:** biologic therapy, phenotype switch, atopic dermatitis, psoriasis, Janus kinase inhibitors

## Abstract

Biologic therapies are highly efficacious for pediatric atopic dermatitis (AD) and psoriasis (PsO); however, their expanding use has led to increased reports of phenotype switching—a paradoxical condition where biologics induce new-onset immune-mediated dermatoses. To address clinical uncertainty regarding these complex reactions, this review evaluates real-world case reports, case series, and observational studies of pediatric AD and PsO patients who developed phenotypic switching during biologic therapy. Dupilumab is the most frequently implicated agent, commonly inducing psoriasiform, pustular, or palmoplantar eruptions in AD patients via a compensatory shift from Th2- to Th17-predominant immunity. Conversely, phenotype switching from IL-12/23 and TNF-α inhibitors remains rare in pediatric dermatology. For severe, refractory cases or AD-PsO overlaps, Janus kinase (JAK) inhibitors demonstrate significant rescue efficacy, provided appropriate baseline and ongoing laboratory monitoring is maintained. Pediatric patients experiencing biologic-induced phenotype switching can be successfully managed in clinical practice through early recognition and the implementation of the practical, evidence-based treatment algorithm proposed in this review.

## 1. Introduction

Phenotype switching represents a challenging category of treatment-emergent adverse events where a biologic agent, typically used to treat a specific immune-mediated inflammatory disease, either triggers the de novo development or the exacerbation of a different inflammatory condition [1,2]. While these reactions were initially identified in adults, they are increasingly recognized in the pediatric population as the use of therapeutic monoclonal antibodies increases [3,4]. In pediatric inflammatory skin diseases, the most common manifestations involve a shift between atopic dermatitis (AD) and psoriasis (PsO), reflecting the complex interplay between different arms of the immune system [1,5,6].

**Figure 1:**
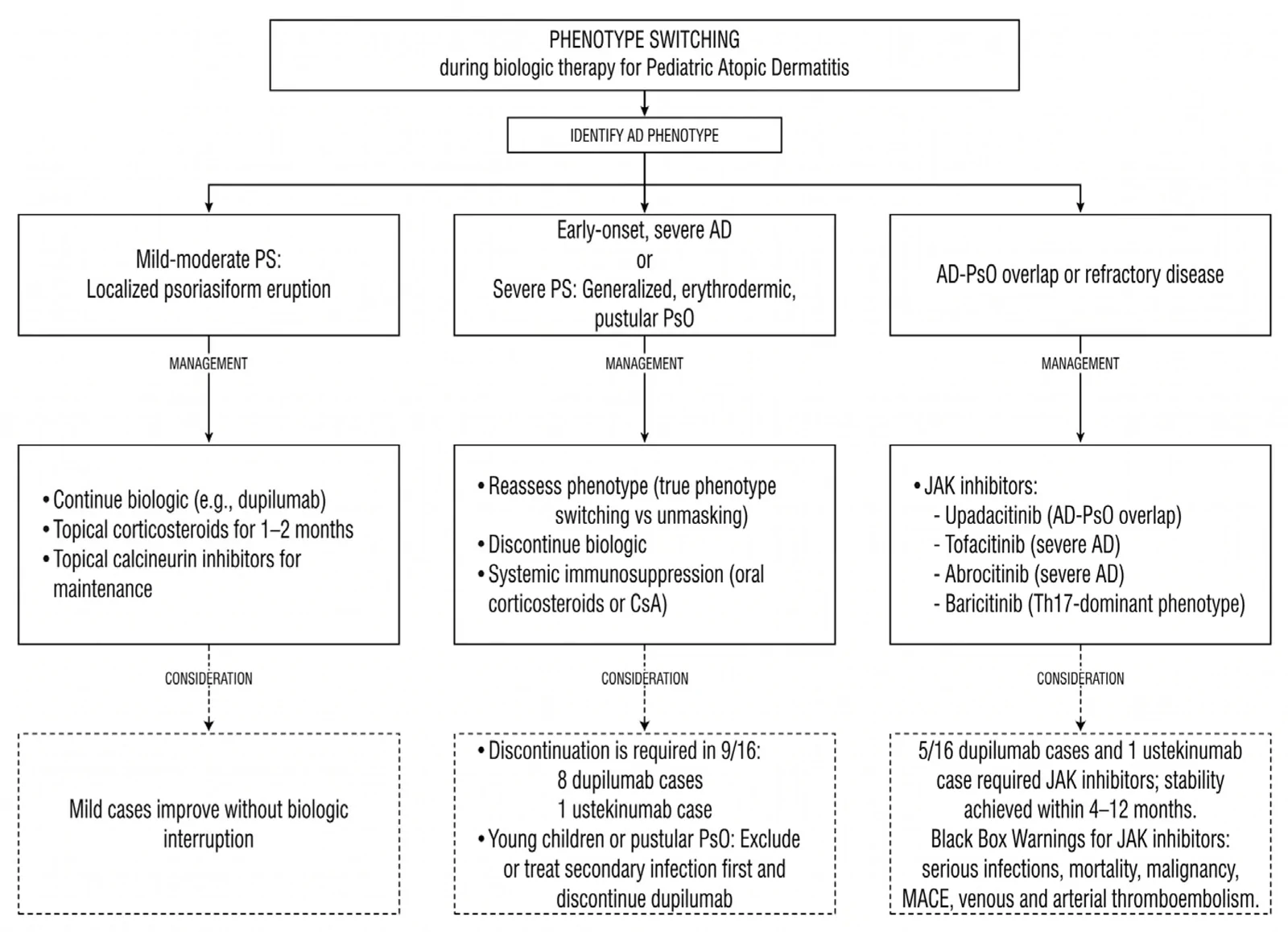
A proposed treatment algorithm for Phenotype Switching during biologic therapy for Pediatric Atopic Dermatitis. Abbreviations: AD, atopic dermatitis; PS, phenotype switching; AD-PsO overlap: Atopic dermatitis–psoriasis overlapping; CsA, cyclosporine; MACE, major adverse cardiovascular events. This is a proposed treatment algorithm for phenotype switching occurring during biologic therapy for atopic dermatitis, based on limited case evidence and expert opinion. The management strategies and considerations summarized in this algorithm are based on previous reports [4,5,6,7,8,9,10,11,12,13,14].

While phenotype switching is increasingly documented in adult cohorts, pediatric data remain limited, primarily consisting of isolated case reports. A significant literature gap exists regarding the systematic evaluation of these paradoxical reactions in children, particularly concerning the management of severe AD-PsO overlapping phenotypes and the application of novel targeted therapies. To address this gap, this review synthesizes current evidence on pediatric phenotype switching across different biologics. Furthermore, it provides an additional perspective by specifically evaluating the emerging role of Janus kinase (JAK) inhibitors as a targeted rescue strategy. By analyzing epidemiologic patterns, underlying immunopathogenesis, and clinical phenotypes, this review aims to equip pediatric dermatologists with practical insights—including a proposed clinical decision-making algorithm (Figure 1).

## 2. Materials and Methods

**Figure 2:**
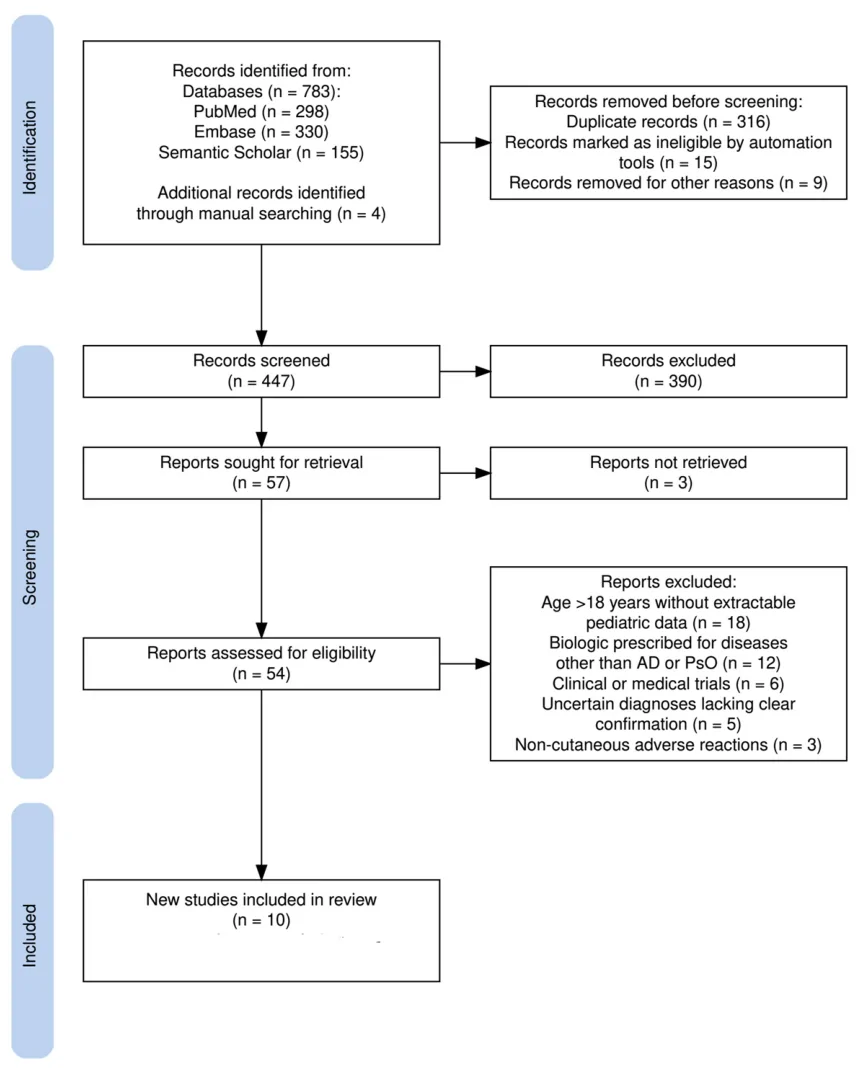
PRISMA flow diagram illustrating the literature search and study selection process.

This review includes studies identified through systematic searches of PubMed, Embase, and Semantic Scholar from database inception to 31 July 2026, by searching “(atopic dermatitis OR psoriasis) AND (biologic therapy OR biologics OR monoclonal antibody) AND (phenotype switch OR paradoxical reaction) AND (pediatric OR children OR infant OR adolescent),” as well as pertinent references by manual searching. Eligible studies included pediatric patients (≤18 years) receiving biologic therapy specifically for AD or PsO, who developed phenotype switching during treatment. Biologic agents collected in this literature review included all biologics used for the treatment of pediatric AD and PsO, including dupilumab, tralokinumab, lebrikizumab, nemolizumab, etanercept, ustekinumab, secukinumab, ixekizumab, guselkumab, and adalimumab, all of which were included in the search strategy. Initially, two independent researchers screened the titles and abstracts to remove duplicates and clearly irrelevant articles using EndNote 20 (Clarivate, London, UK), followed by a full-text review of the remaining records. Real-world case reports, case series and observational studies were included. Studies were excluded if they were clinical or medical trials, if they involved patients older than 18 years without extractable pediatric data, if biologic therapy was prescribed for diseases other than AD or PsO (e.g., inflammatory bowel disease or asthma, or other dermatologic/rheumatologic diseases), or if reported adverse reactions were non-cutaneous, such as systemic hypersensitivity or anaphylaxis. Furthermore, to ensure the reliability of the clinical evidence, cases with “uncertain diagnoses”—defined as those lacking clear clinical features or histopathological confirmation in the medical records—were strictly excluded. The comprehensive literature search, screening, and study selection process is summarized in the PRISMA flow diagram (Figure 2).

An initial systematic search of PubMed, Embase, and Semantic Scholar was conducted, supplemented by additional records identified through manual searching. Prior to screening, records were removed due to duplication, ineligibility determined by automation tools (e.g., animal studies or non-English records), and other reasons such as non-journal publication types, retracted articles, or invalid metadata. Following title and abstract screening, clearly irrelevant studies were excluded. Reports that could not be retrieved—primarily conference abstracts or those with unavailable full texts—were also excluded. The remaining full-text articles were assessed for eligibility, excluding those involving adult patients without extractable pediatric data, alternative biologic indications, trial designs, or uncertain diagnoses. Ultimately, 10 studies comprising 17 well-documented pediatric cases were included in the qualitative synthesis.

## 3. Pathogenesis

**Figure 3:**
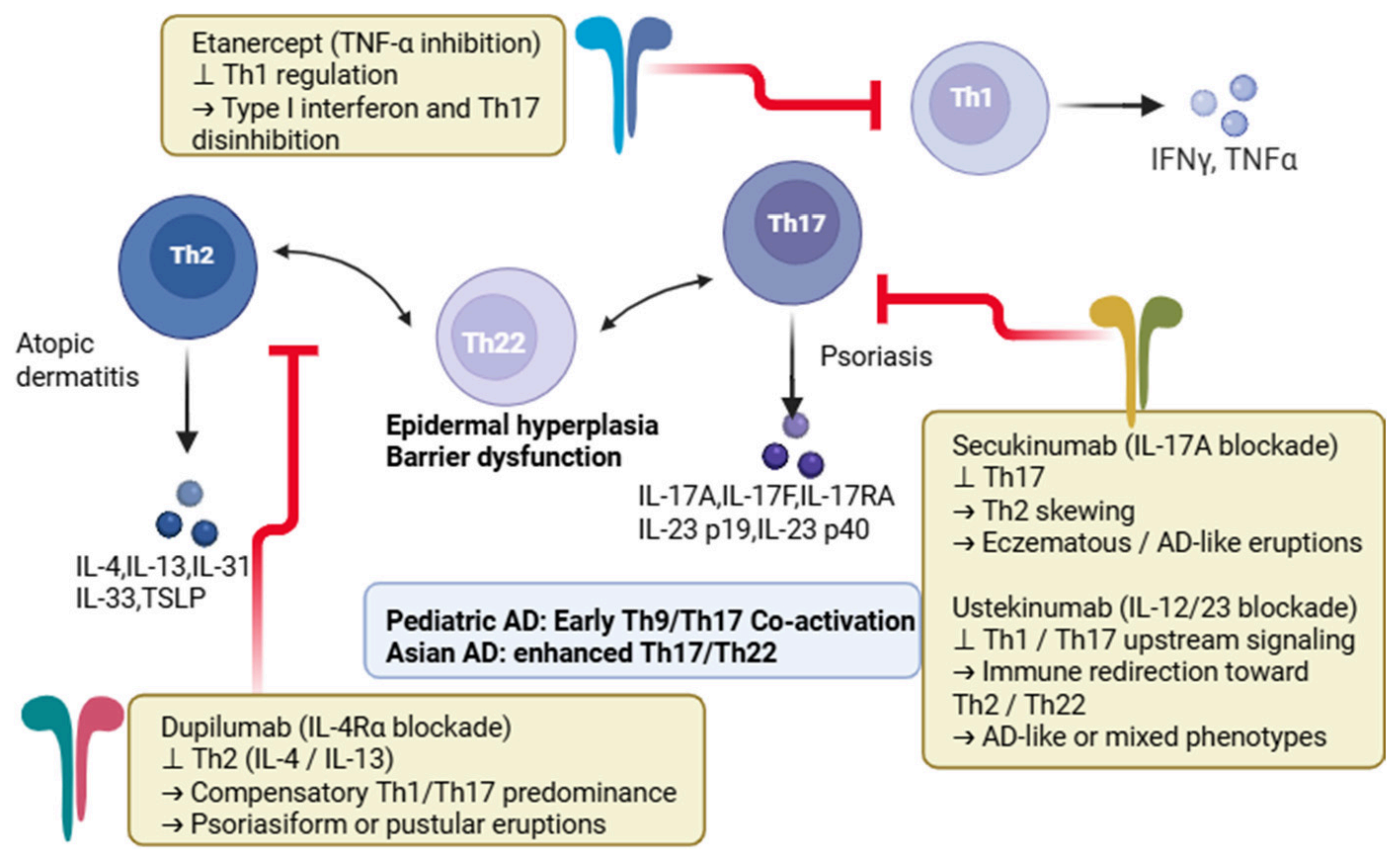
Shared Th2–Th17–Th22 Immunopathogenesis Underlying Biologic-Induced Phenotype Switching in Pediatric Atopic Dermatitis and Psoriasis. In this diagram, red lines with blunt ends indicate the pharmacological inhibition of specific immune pathways by biologic agents. Yellow boxes detail the subsequent mechanisms and clinical outcomes of phenotype switching resulting from this targeted blockade. Solid black arrows represent downstream cytokine production and pathway progression leading to disease manifestation, while curved, bidirectional black arrows denote the immunological crosstalk between different T-helper (Th) cell subsets. Note that this figure exclusively includes FDA-approved biologics for pediatric atopic dermatitis or psoriasis with documented reports of phenotype switching. This figure was created with BioRender.com.

AD and PsO are traditionally regarded as immunologically opposing diseases, dominated by Th2 and Th17/IL-23 pathways, respectively; however, increasing molecular and clinical evidence supports partial overlap of immunogenic pathways (Figure 3). The pathogenesis of phenotype switching is rooted in the disruption of the delicate cytokine balance between different T-helper (Th) cell immune axes [1,2].

### 3.1. T-Cell Polarization Skewing

PsO is primarily a Th1/Th17-driven disorder, while AD is dominated by Th2-mediated immunity [1,7,8], resulting in dysregulation and inflammatory loops in the epithelial immune microenvironment [15]. Inhibiting one pathway can paradoxically upregulate another; for instance, blocking the Th2 pathway with dupilumab can lead to a compensatory shift toward Th1/Th17 predominance, resulting in psoriatic lesions [4,7]. Conversely, blocking the Th17 axis (as seen with IL-17 inhibitors) can skew the immune response toward a Th2 phenotype, inducing eczematous eruptions [5,16].

The widespread use of dupilumab in AD disrupts Th2 signaling by blocking IL-4Rα, a pathway essential for activating STAT6 and maintaining high expression of the master transcription factor GATA3. Because GATA3 actively suppresses alternative lineages—such as by downregulating STAT4 to inhibit Th1 differentiation and repressing Th17-related epigenetic loci—its functional blockade removes critical transcriptional inhibition. this polarization mechanism enforces lineage exclusivity; GATA3 and STAT6 actively silence the expression of Th1 cytokines, strongly suppressing the differentiation of interferon-γ (IFN-γ)-producing Th1 cells while also regulating Th17-driven inflammatory pathways. When biologics such as dupilumab block the IL-4Rα subunit, this Th2-polarizing signaling loop is dismantled and thus disrupting the balance of T-helper cell phenotypes [17]. This enables a compensatory immunological shift toward Th1 and Th17 predominance, mediated by the unopposed actions of T-bet and RORγt, which manifests clinically as psoriasiform or pustular eruptions [18].

IL-4 and IL-13 drive type 2 inflammation via receptor complexes sharing the IL-4Rα subunit. IL-4 acts through Type I and II receptors to promote Th2 differentiation, IgE class switching, and innate cell priming, while IL-13 signals exclusively through Type II receptors to induce tissue-specific pathologies like goblet cell metaplasia [19]. Both cytokines stimulate chemokine release to recruit pathogenic eosinophils. Dupilumab binds the shared IL-4Rα subunit, mechanically blocking both cytokines and halting Type I and Type II receptor signaling. This dual blockade prevents downstream STAT6 phosphorylation, effectively suppressing the type 2 transcriptome and reversing allergic inflammation [20].

### 3.2. Shared Genetic Susceptibility

AD and PsO share activation of the Th22 axis, with IL-22-mediated epidermal hyperplasia and barrier dysfunction [21], and exhibit convergence at specific genomic loci, including chromosome 5q31.1–q33.1, which contains IL-13 and other immune regulatory genes, a cytokine implicated in the pathogenesis of both diseases [5,22,23]. Baurecht et al. demonstrated that this locus represents a Th2 cytokine regulatory region with opposing risk alleles, conferring susceptibility to either AD or PsO depending on allele orientation [22]. Overall, genetic studies largely support AD and PsO as immunogenetically opposing disorders; however, the presence of overlapping susceptibility loci and shared cytokine pathways suggests partial convergence.

### 3.3. Ethnic and Age-Related Differences in Disease Phenotypes

The Asian AD phenotype demonstrates concurrent Th2 and enhanced Th17/Th22 polarization [24], resulting in psoriasiform clinical and histologic features, such as acanthosis and parakeratosis, despite high IgE levels. Transcriptomic analyses place Asian AD between European AD and PsO, highlighting a phenotype and immune spectrum rather than strict dichotomy [21]. These shared and intersecting immune pathways provide a biologic basis for phenotype overlap and variable therapeutic responses to targeted biologics, particularly in pediatric patients [25].

Real-world transcriptomic profiling has challenged mutual exclusivity models. For example, Taiwanese plaque psoriasis lesions demonstrate a distinct dual Th17/type 2 (Th2) transcriptomic endotype. These lesions simultaneously upregulate classical Th17 markers (IL-17A, IL-17C, IL-23A) and Th2 markers (IL-4R, STAT6, CCL17, TSLP), proving that tissue-specific microenvironments can support mixed, dual-polarized immune profiles [26].

Additionally, the baseline skin phenotype of new-onset pediatric AD is substantially different from that of adult AD. Although excess Th2 activation remains a shared hallmark, early and concomitant activation of Th9 and Th17 pathways are highly activated at disease initiation of pediatric AD [27]. Upregulation in IL-19 levels might be a potential molecular link bridging Th2 and Th17 activation [27]. Recognition of this Th2–Th17–Th22 immune continuum regarding the ethnic and age-related disease variations is essential for precision-based management and rational therapeutic selection in pediatric dermatology.

### 3.4. Controversies and Areas of Uncertainty

These complex immunological overlaps and demographic variations introduce significant controversies regarding pediatric phenotype switching. The primary diagnostic challenge lies in distinguishing “true” biologic-induced phenotype switching from the “unmasking” of a pre-existing, subclinical dual-phenotype disease (e.g., an AD-PsO overlap) [4]. Because early-onset pediatric AD intrinsically features higher Th17 and Th9 activity [28], it remains debated whether biologics actively create a new immune shift or simply unmask a pre-existing condition. Furthermore, it remains unclear why current literature shows East Asian pediatric patients are disproportionately affected by dupilumab-induced reactions. This trend might originate from a true genetic susceptibility, given the higher baseline Th17/Th22 activation in Asian AD [21,24]. Alternatively, it could simply be an artifact of regional prescribing habits, healthcare policies, or publication bias.

## 4. Epidemiology

Available evidence indicates that phenotype switching reactions to biologic therapies in pediatric dermatology remain rare but increasingly recognized, largely reflecting the growing use of targeted immunomodulatory agents in children. To date, the literature consists predominantly of isolated case reports and small case series, with only 17 well-documented pediatric cases (16 dupilumab cases, 1 ustekinumab case) reported across all biologic classes (Table 1).

Reported pediatric age range from 4 to 18 years, with a concentration in school-aged children and adolescents. Ethnically, East Asian patients are disproportionately represented in dupilumab-associated phenotype switching, especially psoriasiform and pustular phenotypes, while reports from Europe and North America are fewer but include similar clinical patterns. This apparent ethnic skew may reflect genetic susceptibility—the inherently higher baseline Th17 and Th22 axis activation observed in Asian populations—rather than true, intrinsic population-level risk differences, confounded by different environmental exposures, regional health insurance reimbursement policies, and varying healthcare-seeking behaviors.

Among biologics, dupilumab (IL-4/IL-13 receptor blockade) is by far the most frequently reported agent in phenotype switching induced by biologics during treatment for pediatric AD and PsO. Other biologic classes are far less commonly reported in the pediatric population such as IL-12/23 inhibition (ustekinumab).

## 5. Evidence in Pediatric Patients

### 5.1. Dupilumab

Among biologic therapies, dupilumab is the most frequently reported agent associated with phenotype switching in pediatric inflammatory skin diseases. To date, only 16 pediatric cases have been documented—predominantly as case reports or small case series—with East Asian patients disproportionately represented. Most cases describe psoriasiform, pustular, or palmoplantar psoriasis-like eruptions emerging despite good control of underlying AD. Reported time to onset ranges from 1 week to 2 years, and 7 of 16 cases involved early-onset, long-standing severe AD.

Parker et al. [4] presented a retrospective case series of six pediatric patients (aged 4–18 years) with moderate-to-severe AD treated with dupilumab. New-onset psoriasiform dermatitis developed at a median of 8 months after dupilumab initiation, despite substantial AD improvement. Lesions predominantly involved typical psoriatic sites and were manageable with topical corticosteroids, allowing dupilumab continuation in 5 cases (83%). Only one child required treatment discontinuation, while another had pre-existing PsO unmasked by effective AD control [4]. Dupilumab-induced inverse psoriasis was also observed in two 13-year-old heart transplant recipients after 6 months and 2 years of dupilumab, respectively, despite concomitant systemic immunosuppression with tacrolimus and azathioprine [6]. In both patients, phenotype switching reactions were effectively managed using topical corticosteroids and calcineurin inhibitors without the need to discontinue dupilumab [6]. In a case requiring dupilumab discontinuation, a 16-year-old boy with severe AD developed psoriasis plaques on his arms, neck, face, and scalp 4 weeks after starting therapy. Since both his AD and PsO worsened by week 12 despite topical clobetasol, dupilumab was discontinued and replaced with oral cyclosporine (3 mg/kg/day), which successfully cleared the PsO and improved the AD within 4 weeks [9].

**Figure 4:**
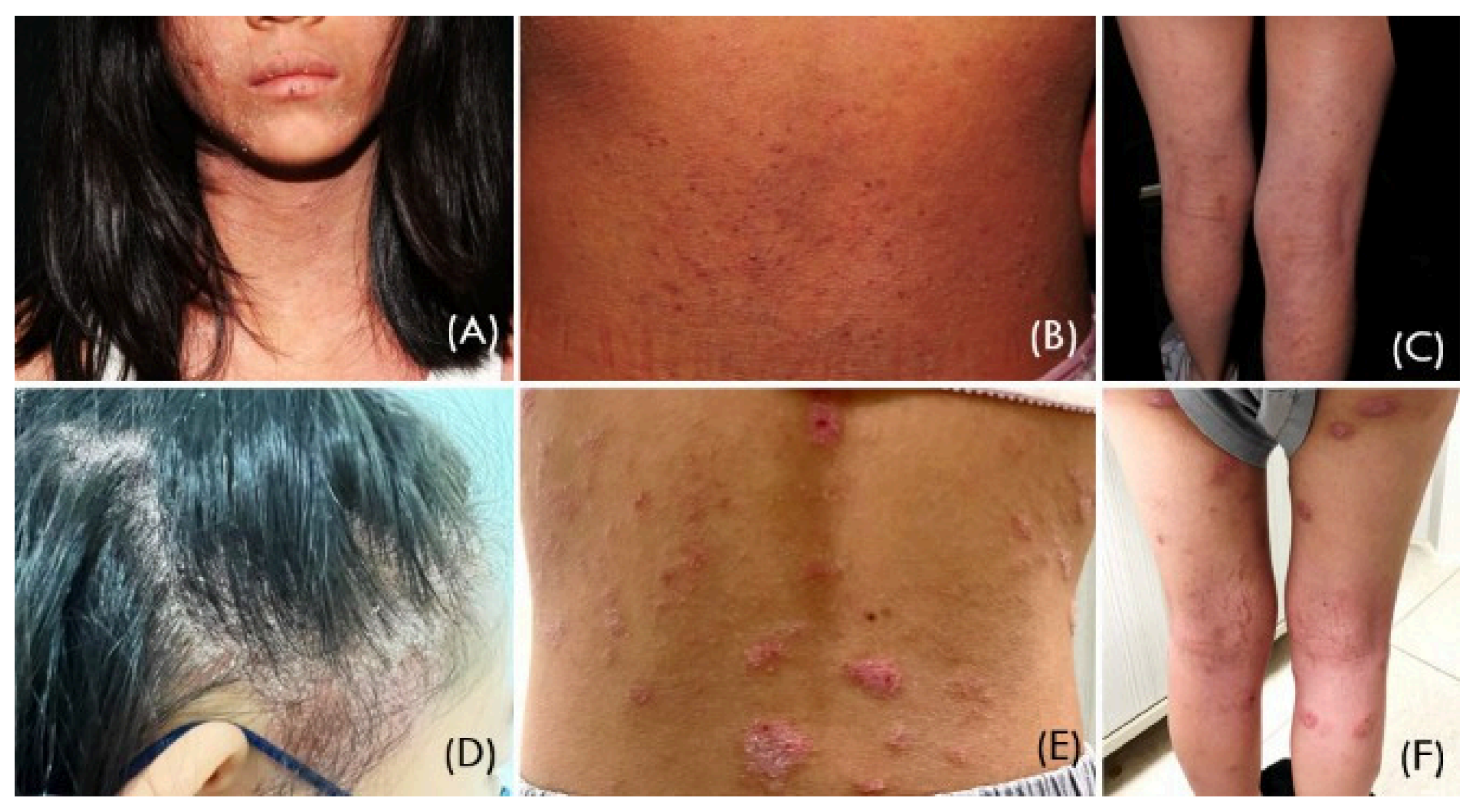
Dupilumab-Induced Psoriasiform reaction in pediatric atopic dermatitis. (**A**–**C**) Cutaneous manifestations of atopic dermatitis before dupilumab therapy. (**D**–**F**) Following 14 doses of dupilumab (300 mg Q2W), the patient developed diffuse psoriasiform plaques one week after 7 months.

JAK inhibitors have emerged as an effective therapeutic strategy to resolve these paradoxical and sometimes refractory reactions. A 10-year-old East Asian girl developed psoriasiform plaques one week after the 14th dose of dupilumab; disease was refractory to biologic switching (secukinumab) and cyclosporine but achieved sustained remission with upadacitinib over one year [7] (Figure 4). Two 17-year-old Chinese monozygotic twins developed psoriasiform erythema 20 weeks after dupilumab, despite achieving Eczema Area and Severity Index-75 (EASI-75) earlier; lesions showed marked IL-17A overexpression, and baricitinib led to significant improvement for over 24 weeks [10]. Similarly, a 15-year-old Asian boy who developed widespread psoriasiform dermatitis after 8 months of dupilumab achieved near-complete resolution of PsO and maintained AD control within 4 weeks of switching to abrocitinib, with sustained efficacy over 6 months [11].

Pustular psoriasiform reactions have been described in younger children. A 4-year-old Chinese boy (weighing 26 kg) with severe AD developed pustular PsO one week after an off-label dupilumab loading dose (600 mg), suggesting a dose-dependent rapid onset. The eruption progressed despite drug withdrawal and systemic corticosteroids, but completely resolved within two weeks of oral thiamphenicol (a chloramphenicol-analogue antibiotic empirically used in East Asia for respiratory and genitourinary infections), without recurrence over one year [8]. Similarly, a 13-year-old Asian boy developed acute generalized pustular PsO 10 days after dupilumab (400 mg), resolving rapidly with drug discontinuation alone [12]. Palmoplantar involvement remains rare but was reported in a 17-year-old East Asian boy with early-onset severe AD who developed psoriasiform lesions on previously unaffected palms and soles 2 months after starting dupilumab. Symptoms improved following drug withdrawal and a 2-month cyclosporine course [13].

Psoriatic reactions to dupilumab in children are clinically heterogeneous, with onset ranging from days to months and phenotypes spanning psoriasiform, pustular, and palmoplantar disease. While some cases are mild and manageable without drug cessation, severe or refractory presentations—particularly in early-onset, long-standing AD—may require prompt discontinuation and alternative systemic therapy, including JAK inhibition, highlighting the need for early recognition and individualized management.

### 5.2. Secukinumab

Secukinumab is a biologic that targets the IL-17A cytokine and is approved by the EMA and FDA for psoriasis in children 6 years of age and older. Although pediatric cases have not yet been reported, phenotype switching induced by IL-17 inhibition was documented in a 20-year-old adult with severe PsO who developed generalized eczema two months after biologic therapy [29].

### 5.3. Ustekinumab

Evidence regarding ustekinumab-induced phenotype switching in pediatric and adolescent inflammatory skin disease remains extremely limited.

In the case reported by Gargiulo et al. [14], a 12-year-old Italian boy with PsO since early childhood (baseline Psoriasis Area Severity Index [PASI] 10) and concomitant AD (EASI 12) was initially treated with ustekinumab 45 mg for 18 months. Owing to the predominance of AD lesions, therapy was switched to dupilumab. Although AD improved, the patient experienced a relapse of PsO involving the nails and soles approximately 4 months after the phenotype switch. Dupilumab was discontinued, and upadacitinib 15 mg/day was initiated. After 16 weeks of treatment, the patient achieved complete remission of both PsO and AD, showing favorable short-term follow-up outcomes.

This represents a case of AD–PsO overlap since early childhood, in which biologic switching alone was insufficient, and highlights upadacitinib after biologic discontinuation as an effective rescue strategy. Notably, this represents one of the 6 pediatric cases identified in our systematic search requiring JAK inhibitor initiation, emphasizing the role of JAK inhibition in refractory overlap phenotypes.

### 5.4. TNF-α Inhibitor

Reviewing previously reported phenotype switching induced by TNF-α inhibitors in pediatric patients, most reactions manifest as psoriasiform eruptions, despite TNF-α inhibitor (etanercept, adalimumab) being approved therapies for PsO. Notably, in pediatric patients, TNF-α inhibitors are predominantly prescribed for non-dermatologic inflammatory diseases, including juvenile idiopathic arthritis (JIA), inflammatory bowel disease (IBD), and chronic nonbacterial osteomyelitis (CNO) [30,31]. A multicenter retrospective cohort study of 103 pediatric patients receiving TNF-α inhibitor—primarily for IBD—demonstrated that psoriasiform eruptions typically occurred after prolonged exposure (median latency 14.5 months) and often involved multiple anatomical sites. Although 37% discontinued the initial TNF-α inhibitor, the majority (83%) were ultimately able to continue TNF-α inhibitor therapy with adjunctive dermatologic management or intra-class switching [3]. Through literature searching, no pediatric cases of phenotype switching have been identified during TNF-α inhibitor treatment for PsO.

## 6. Management Strategies

Management strategies for phenotype switching are heterogeneous and individualized, reflecting variations in severity and underlying disease control. Initially, the severity and specific phenotype switching must be assessed. For mild, localized psoriasiform eruptions, the primary strategy involves maintaining the current biologic therapy while initiating topical corticosteroids or calcineurin inhibitors, as most cases stabilize without biologic interruption [4,6,32]. In contrast, severe, refractory, generalized pustular psoriasis or erythrodermic flares frequently necessitate biologic discontinuation and escalation to systemic immunomodulatory therapy. In these severe scenarios, particularly in young children, clinicians must first explicitly rule out secondary cutaneous infections before initiating systemic immunosuppressants. Given that the majority of reported cases involve AD, a treatment algorithm based on current case evidence and expert opinion is proposed to assist pediatric dermatologists in clinical decision-making when phenotype switching occurs during biologic therapy (Figure 1). It is crucial to emphasize that this algorithm serves as a practical, expert-informed framework derived from real-world case evidence, rather than validated pediatric guidelines. All decisions regarding biologic switching or JAK inhibitor initiation must be highly individualized, weighing the patient’s baseline disease severity against the safety profiles of the rescue therapies.

Special considerations are warranted in the management of phenotype switching in pediatric patients. In young children and in cases presenting with pustular or rapidly progressive PsO, heightened vigilance is required. Secondary cutaneous infections should be actively excluded or treated, and prompt withdrawal of dupilumab is recommended. Notably, biologic switching alone (e.g., from dupilumab to ustekinumab or secukinumab) has demonstrated limited efficacy in pediatric AD-associated phenotype switching. JAK inhibitors (e.g., upadacitinib, abrocitinib, baricitinib) have emerged as a consistent and effective rescue strategy for pediatric cases with overlapping AD–PsO phenotypes, achieving rapid improvement and sustained remission over follow-up periods ranging from 16 weeks to 1 year. Furthermore, treatment-emergent eczematous reactions induced by IL-17 and IL12/23 inhibitors have also been successfully be managed with JAK inhibitors [29,33,34,35,36].

Although long-term data remain scarce, available follow-up suggests that most pediatric phenotypic switching is clinically manageable and non-life-threatening, with no reported mortality and limited irreversible sequelae. Recurrence is possible but is often controllable with intermittent therapy, highlighting the importance of early recognition, careful phenotype assessment, and flexible treatment adjustments.

### 6.1. Upadacitinib

Upadacitinib, a selective inhibitor preferentially targeting JAK1, is approved for both AD and PsA in adolescents and adults. Upadacitinib suppresses IL-6, IL-15, and interferon-α/γ production, and inhibits STAT3 activation, which in turn promotes keratinocyte differentiation and restores filaggrin, loricrin, and natural moisturizing factor expression—addressing barrier dysfunction common to both AD and psoriasis [37]. AD-PsO overlapping phenotypes are distinctly driven by the co-existence of Type 2 (Th2) and Type 3 (Th17) inflammation, intrinsically linked to JAK-STAT signaling pathways. While traditional biologics targeting a single inflammatory axis (either solely AD-targeted or PsO-targeted) are often inadequate for these overlapping phenotypes, JAK1 inhibitors effectively suppress this dual inflammation [38].

In Phase III clinical trial programs involving adolescents, upadacitinib demonstrated high efficacy in reducing the signs and symptoms of AD and improving quality of life, characterized by a rapid onset of action. While the higher dose (30 mg daily) demonstrated the highest efficacy at reducing EASI scores up to 16 weeks—showing superiority to dupilumab in head-to-head clinical trials—the lower dose (15 mg daily) still provides excellent clinical improvement [39]. However, it is crucial to note that pediatric patients exhibit a higher frequency of blood and lymphatic adverse events (particularly cytopenias) and an elevated risk of infectious adverse events when treated with upadacitinib compared to adult populations [40]. Therefore, vigilant patient selection and strict adherence to laboratory monitoring protocols are paramount when utilizing upadacitinib as a rescue therapy for phenotype switching.

### 6.2. Abrocitinib

Abrocitinib is a selective oral JAK1 inhibitor, EMA- and FDA-approved only for refractory, moderate-to-severe AD in patients ≥ 12 years old [39]. JAK1 lies downstream not only of the IL-4/IL-13 receptors driving Th2 disease, but also of multiple cytokines involved in Th17/IL-23-mediated inflammation. By blocking JAK1 selectively, abrocitinib interrupts signaling for IL-4, IL-13, IL-31, and TSLP (Th2/pruritus axis driving AD) as well as IFN-γ and IL-22 signaling, cytokines also implicated in psoriasiform inflammation, without inhibiting JAK2-dependent pathways [5,11,41].

Rather than blocking only one arm as dupilumab does, this dual suppression makes abrocitinib a highly rational treatment strategy for patients who develop PsO on dupilumab, since it can control both phenotypes simultaneously. In head-to-head data, abrocitinib 200 mg/day demonstrated greater and more rapid AD clearance and pruritus relief than dupilumab 300 mg, with both treatments converging in efficacy over time [39]. In terms of safety and treatment guidelines, it is crucial to note that the use of abrocitinib should not be combined with other JAK inhibitors or systemic immunosuppressants [39].

### 6.3. Baricitinib

Baricitinib preferentially inhibits both JAK-1 and JAK-2 and is an effective systemic therapy for AD. In Europe, it is approved for the treatment of moderate-to-severe AD in patients aged 2 years and older. While it is approved and available in the US for other immune-mediated conditions, it is currently used off-label for AD.

Baricitinib’s dual JAK1/JAK2 inhibition simultaneously blocks type 2 (Th2) and type 3 (Th17/Th22-associated) inflammatory pathways, making it effective for AD-PsO overlap and phenotype switching. By inhibiting JAK1/JAK2-dependent cytokines including IL-6 and IFN-γ, as well as JAK1/JAK3, JAK1/TYK2, and JAK2/TYK2-dependent signaling with comparable potencies in human leukocytes [42], baricitinib broadens coverage beyond JAK1-selective agents. This suppresses AD-driving signals (IL-4, IL-13, IL-31, TSLP) and psoriasis-relevant pathways like IFN-γ and Th1/Th17-adjacent axes [41,43].

### 6.4. Recommended Monitoring for JAK Inhibitors

#### 6.4.1. Baseline and Routine Laboratory Monitoring

The FDA recommends checking a complete blood count (CBC) with differential and liver enzymes at baseline, and again after initiation or dose escalation. Specific monitoring intervals vary by agent—abrocitinib requires re-checking at 4 weeks, whereas upadacitinib monitoring follows routine clinical management after baseline [39]. For upadacitinib, pediatric labels advise checking CBC with differential and liver enzymes at baseline, followed by routine management without a fixed re-check interval specified [39].

Pediatric patients require vigilance regarding blood and lymphatic adverse events, as cytopenias (specifically lymphopenia, neutropenia, and anemia) are more frequently reported in this demographic than in adults [39]. Furthermore, in the pediatric Phase 1 trial for polyarticular-course juvenile idiopathic arthritis (SELECT-YOUTH), laboratory abnormalities were consistent with the known adult safety profile. Hepatic disorders, neutropenia, and elevated creatine phosphokinase (CPK) were among the most common findings associated with upadacitinib, particularly at higher doses [44,45]. Other essential baseline testing for all oral JAK inhibitors includes screening for viral hepatitis, tuberculosis, and pregnancy [39].

Additionally, clinicians should evaluate renal function at baseline, particularly when considering baricitinib, as dose adjustments are required for patients with renal impairment. Population pharmacokinetic data in children show that renal clearance and body weight significantly influence baricitinib exposure, similar to adults, supporting weight- and eGFR-based dosing proposals [46].

#### 6.4.2. Lipid Profiles

Dyslipidemia, including elevated low-density lipoprotein (LDL), occurs commonly enough across the JAK inhibitor class to warrant periodic monitoring. Dose adjustments or medication discontinuation may be required if lipid abnormalities become clinically significant [46]. Lipid panels should be evaluated at baseline and after treatment initiation, specifically at 4 weeks for abrocitinib and 12 weeks for upadacitinib [39].

#### 6.4.3. Infection Risk

Infection risk remains the most consistently reported safety signal for JAK inhibitors across all age groups. Pharmacovigilance data show both pediatric and adult patients have increased reporting of infections and gastrointestinal adverse events [41]. Real-world pharmacovigilance analysis utilizing the FDA Adverse Event Reporting System (FAERS) database revealed distinct, age-dependent infectious adverse event (iAE) profiles: pediatric patients (0–14 years) show an elevated iAE risk with abrocitinib and upadacitinib. The most frequently reported infections include COVID-19, herpes zoster, upper respiratory tract infections, and pneumonia [45,47,48,49].

To mitigate these risks, the American Academy of Allergy, Asthma & Immunology (AAAAI/ACAAI) guidelines mandate baseline screening for latent tuberculosis, viral hepatitis, and other chronic infections prior to initiating oral JAK inhibitor therapy [44]. Crucially, pediatric dermatologists must also ensure that all age-appropriate immunizations are up to date prior to JAK inhibitor initiation, keeping in mind that live vaccines are contraindicated during active treatment [46,50].

#### 6.4.4. Major Adverse Cardiovascular Events (MACE) and Malignancies

While the risk of major adverse cardiovascular events (MACE) and malignancies prompted FDA black box warnings for the JAK inhibitor class, these events remain exceedingly rare in pediatric populations; however, judicious patient selection remains essential. In an analysis of FAERS and Canada Vigilance data, the combined proportion of boxed-warning-associated adverse events (serious infection, mortality, malignancy, adverse cardiovascular events, thrombosis) was lower in children than adults for most agents, though baricitinib showed comparable rates (8.2% pediatric vs. 8.8% adult) [40]. A separate pediatric-focused review similarly concludes that existing literature suggests an acceptable safety profile for JAK inhibitors in children, while emphasizing the need for continued long-term vigilance given the limited pediatric dataset [51].

## 7. Limitations

There are several important limitations in this review. Most notably, the available pediatric data regarding biologic-induced phenotype switching derive almost exclusively from individual case reports and small case series. As a result, the true incidence or frequency of these paradoxical reactions in the pediatric population cannot be accurately established. Therefore, interpretations concerning potential risk factors, ethnic patterns, and long-term treatment outcomes must be approached with appropriate caution. The proposed clinical algorithm, while practically useful, should be viewed as an expert-guided framework rather than a validated guideline. Future large-scale, prospective clinical trials and real-world pharmacovigilance studies are needed to overcome these limitations and provide robust, pediatric-specific safety profiles.

## 8. Conclusions

Phenotype switching is an increasingly recognized adverse event in pediatric dermatology. It is most frequently triggered by dupilumab, which drives a compensatory Th2- to Th17 immune shift, whereas reactions to IL-12/23 and TNF-α inhibitors remain uncommon. In severe or refractory cases, particularly those with overlapping atopic dermatitis and psoriasis features, JAK inhibitors demonstrate significant rescue efficacy, provided that rigorous laboratory monitoring is maintained. Ultimately, the integration of advanced biomarker characterization with robust prospective and mechanistic studies is essential to refine risk stratification. Together, these efforts will drive the implementation of highly precise, personalized treatment paradigms for the use of targeted therapeutics in pediatric dermatology.

## Figures and Tables

**Table 1:** Summary of Phenotype switching following biologic agents in Pediatric Atopic Dermatitis and Psoriasis.

Author	Patient Profile (Baseline Disease, N, Age)	Dosing	Phenotype Switching	Time to Onset	Drug Withdrawal (Case Number)	Management (Case Number)	Long-Term Data (Case Number)
Dupilumab							
Parker et al. (2021) [4]	Moderate-to-severe AD N = 6; median 13 years	weight-based dosing 150–300 mg Q2W	PsO	Median 8 months (range 6–12 months)	N (5/6)	Ustekinumab (1, ineffective); Medium- to high-potency topical corticosteroids for 1–2 months (3); dupilumab continued (5); tofacitinib and IVIG (1)	Clearance or near-clearance (5); some recurrences controlled with intermittent topical corticosteroids up to 8–18 months (5)
Lockard et al. (2024) [6]	AD N = 2; 13 years	Not specified	Inverse PsO	6 months (pt 1); 2 years (pt 2)	N (2/2)	Topical corticosteroids BID (2); tacrolimus 0.03% ointment for maintenance (2)	Full control (pt 1); nearly complete improvement (pt 2)
Tsai and Yu (2025) [7]	AD N = 1; 10 years	300 mg Q2W	PsO	7 months	Y (1/1)	Alternating secukinumab and dupilumab plus CsA 100 mg/day, then upadacitinib 15–30 mg/day	Stable for 1 year with occasional mild flare-ups on upadacitinib
Dang et al. (2024) [8]	Early-onset AD (3-year history) N = 1; 4 years	600 mg followed by 300 mg Q4W	Pustular PsO	1 week	Y (1/1)	topical or oral corticosteroids and antihistamines ineffective; oral thiamphenicol 0.25 g BID effective	Near-complete resolution within 2 weeks of thiamphenicol; no recurrence during >1-year follow-up
Trave et al. (2023) [9]	Early-onset AD N = 1; 16 years	600 mg followed by 300 mg Q4W	Plaque PsO	4 weeks	Y (1/1)	Topical clobetasol initially; later switched to cyclosporine 3 mg/kg/day	Disappearance of PsO and improvement of AD within 4 weeks
Ali et al. (2022) [10]	AD since childhood; positive FH of atopy N = 2; 17 years	300 mg Q2W	PsO	20 weeks	Y (2/2)	initiation of baricitinib 2 mg BID	Significant improvement after 16 weeks of baricitinib; no adverse events during 24-week follow-up
Theodosiou et al. (2026) [11]	Severe childhood-onset AD N = 1; 15 years	600 mg followed by 300 mg Q4W	Plaque PsO	8 months	Y (1/1)	initiation of abrocitinib 200 mg QD	Near-complete resolution within 4 weeks; sustained improvement and AD control during 6-month follow-up
Liu et al. (2023) [12]	Severe AD (3-year history); AR (6-year history); positive FH of atopy N = 1; 13 years	400 mg (loading dose)	Pustular PsO	10 days	Y (1/1)	Supportive therapy with topical emollients and anti-allergic treatment	Clinical improvement of erythema and pruritus; no recurrence of pustules during follow-up
Park et al. (2022) [13]	Early-onset, severe AD N = 1; 17 years	600 mg followed by 300 mg Q2W	PsO (palms and soles, mostly in previously AD-unaffected sites)	2 months	Y (1/1)	CsA for 2 months	Not specified
Ustekinumab							
Gargiulo et al. (2023) [14]	AD-PsO overlap since early childhood; PASI 10 N = 1; 12 years	Ustekinumab 45 mg for 18 months; switch to dupilumab 300 mg due to predominance of AD (EASI 12)	Improvement in AD but PsO relapse (nails and soles)	After 4 months of dupilumab switch	Y (1/1)	Upadacitinib 15 mg/day for 16 weeks after biologic discontinuation	After 16 weeks, the patient experienced complete remission of both diseases

Abbreviations: AD, atopic dermatitis; Q2W, every 2 weeks; CsA, cyclosporine; Q4W, every 4 weeks; PsO, Psoriasis; IVIG, Intravenous immunoglobulin; AR, allergic rhinitis; FH, family history; AD-PsO overlap: Atopic dermatitis–psoriasis overlapping; PASI; Psoriasis Area and Severity Index; EASI, Eczema Area and Severity Index.

## Data Availability

No new data were created or analyzed in this study. Data sharing is not applicable to this article.
